# Behavioral risk factors for overweight in early childhood; the ‘Be active, eat right’ study

**DOI:** 10.1186/1479-5868-9-74

**Published:** 2012-06-15

**Authors:** Lydian Veldhuis, Ineke Vogel, Carry M Renders, Lenie van Rossem, Anke Oenema, Remy A HiraSing, Hein Raat

**Affiliations:** 1 Department of Public Health, Erasmus MC – University Medical Centre Rotterdam, Dr Molewaterplein 50, PO Box 2040, 3000 CA, Rotterdam, The Netherlands; 2 Institute of Health Sciences, Faculty of Earth and Life Sciences, VU University Amsterdam, Amsterdam, The Netherlands; 3 Department of Public and Occupational Health, EMGO Institute for Health and Care Research, VU University Medical Centre, Amsterdam, The Netherlands

**Keywords:** Preschool child, Overweight, Obesity, Lifestyle

## Abstract

**Background:**

The lifestyle-related behaviors having breakfast, drinking sweet beverages, playing outside and watching TV have been indicated to have an association with childhood overweight, but research among young children (below 6 years old) is limited. The aim of the present study was to assess the associations between these four behaviors and overweight among young children.

**Methods:**

This cross-sectional study used baseline data on 5-year-old children (n = 7505) collected for the study ‘Be active, eat right’. Age and sex-specific cut-off points for body mass index of the International Obesity Task Force were used to assess overweight/obesity. Multivariable logistic regression analyses were applied.

**Results:**

For children whom had breakfast <7 days/week and watched TV >2 hours/day, the odds ratio (OR) for having overweight (obesity included) was, respectively, 1.49 (95% confidence interval (CI): 1.13-1.95), and 1.25 (95% CI: 1.03-1.51). There was a positive association between the number of risk behaviors present and the risk for having overweight. For children with 3 or all of the risk behaviors having breakfast <7 days/week, drinking sweet beverages >2 glasses/day, playing outside <1 h/day, watching TV >2 hs/day, the OR for overweight was 1.73 (95% CI: 1.11-2.71) (all models adjusted for children’s sex and sociodemographic characteristics).

**Conclusion:**

Given the positive association between the number of behavioral risk factors and overweight, further studies are needed to evaluate the effectiveness of behavioral counseling of parents of toddlers in preventing childhood overweight. In the meantime we recommend physicians to target all four behaviors for counseling during well-child visits.

## Background

Over the last few decades, an epidemic of childhood overweight and obesity occurred worldwide[1-5]. Nearly 43 million children under the age of 5 years were overweight globally in 2010[4]. An important step in successful prevention in pediatrics is the identification of modifiable risk factors of childhood overweight; such risk factors may be important targets for counseling of parents during well-child or pediatric visits to contribute to the prevention of overweight and obesity[6-8]. Overweight is caused fundamentally by an imbalance between energy intake and energy expenditure[1,4,9]. The lifestyle-related behaviors having breakfast, drinking sweet beverages, playing outside and watching TV have been indicated to have an association with childhood overweight[10-14]. The research in which these associations were investigated, included mainly school-aged children. Research among younger children (below 6 years old) is limited[8,15,16]. In studies that did include this young age-group, associations that were found were unclear due to conflicting results and differences in methodology between studies, for example differences in measurement of behavior and adjustment for confounders[8,16-27]. Furthermore, the analyses that were used were primarily simple or bivariate, while risk factors are likely to interact with each other[6,7]. Therefore, more research in early childhood is needed in which large study populations are included[15], and in which multiple behavioral risk factors for childhood overweight are investigated.

The aim of the present study was to assess the associations between the four lifestyle-related behaviors having breakfast, drinking sweet beverages, playing outside and watching TV, and overweight in a large sample of 5-year-old children. In addition, as it is likely that the risk behaviors coexist, the association between the number of risk behaviors that is present and overweight (obesity included) was investigated.

## Methods

### Design and study population

The present cross-sectional study was embedded in the ‘Be active, eat right’ study, which aims to assess the effects of an overweight prevention protocol, as described in detail elsewhere[28]. The Medical Ethics Committee of Erasmus MC University Medical Centre Rotterdam approved the study protocol. A total of 13 638 parents of 5-year-olds were invited by mail for a well-child visit (which has an attendance rate of 95%) [29] at one of the nine municipal health services across the Netherlands participating in the study. These parents were also invited to participate in the ‘Be active, eat right’ study, 64.4% of whom provided written informed consent. Baseline data were collected during the 2007–2008 school year, and these data were used for the present study. Parents completed a questionnaire with items on demographic, socioeconomic, and lifestyle-related characteristics of themselves and their child. Height and weight of the children were measured by trained health care professionals during well-child visits.

Data of children and their parents were excluded from analyses when data were missing on height or weight of the child (n = 34), sex or age of the child (n = 107), ethnicity of the child (n = 75), the lifestyle-related behaviors having breakfast, drinking sweet beverages, playing outside or watching TV by the child (n = 933), sex or age of the parent (n = 13), educational level of the parent (n = 49), employment status of the parent (n = 52) or single parenting (n = 16). After exclusion for these reasons, a study population of n = 7505 remained.

### Lifestyle-related behaviors

We obtained information on the four lifestyle-related behaviors of the children by a questionnaire completed by the parents. The parents reported, for an average week, how many days per week their child has breakfast, the number of sweet beverages (i.e. lemonade, soda, carbonated soda, fruit juice, sugar sweetened dairy products, etc.) their child drinks per day, the duration of outdoor playing time of their child per day, and the amount of time their child watches TV per day. Risk behaviors were defined as having breakfast <7 days/week, drinking sweet beverages >2 glasses/day, playing outside <1 hour/day and watching TV >2 hours/day. The definitions of these risk behaviors are based on international recommendations[11-13,30-32].

### Weight status of the children

Body weight and height of the children were measured by trained health care professionals during well-child visits using standardized methods as described in a protocol[33]. Body weight was measured to the nearest 0.1 kg and height to the nearest 0.1 cm. Body mass index (BMI) was calculated by dividing weight (in kilograms) by height (in meters) squared. The weight status of the children was assessed according to the age and sex-specific cut-off points for BMI as published by the International Obesity Task Force[34]. When the BMI value of a child was the same as or higher than the cut-off point for overweight or obesity for the child’s age and sex, the child was defined as having overweight or obesity.

### Sociodemographic characteristics

We obtained information on sociodemographic characteristics by a questionnaire completed by the parents. We considered the sociodemographic characteristics sex and ethnicity (Dutch, non-Dutch) of the child, educational level (high level, mid level, low level) of the parent, employment status (employed full-time or part-time, not employed) of the parent, and single parenthood (two-parent families, single-parent family or otherwise specified) potential confounders in the association between the behaviors and having overweight or obesity of the children[15]. A child was considered to be of non-Dutch ethnicity when at least one of the parents was born abroad, as defined by Statistics Netherlands[35]. Educational level of the parent was recoded in three categories, according to the Dutch standard classification as defined by Statistics Netherlands[36]: high level (academic higher education/university education, higher professional education), mid level (pre-university education, senior general secondary education, and senior secondary vocational education), and low level (preparatory secondary vocational education, lower secondary vocational education, primary education, and no education).

### Statistical analysis

We examined mean and frequency differences of the sociodemographic characteristics of the parents and their children, and children’s behaviors, between the groups of children with and without overweight (obesity included) using t tests for continuous variables and Chi-square statistics for categorical variables. We used multivariable logistic regression analyses to test the associations between the child’s behaviors and overweight (obesity included) of the child, and we obtained odds ratios (ORs) and 95% confidence intervals (CIs).

In the first model we investigated the associations between each behavior individually and overweight of the children. In the second model we included all four behaviors of the children. Further, we estimated the odds of having overweight (obesity included) associated with having one, two, or three or all of the risk behaviors relative to having none of the risk behaviors. All analyses were adjusted for sociodemographic characteristics.

We examined whether there was interaction between sex and the behaviors in the association with overweight (obesity included) among the children. However, we found no effect modification, so stratification of the analyses was not necessary, but we adjusted all models for sex of the child. We performed the statistical analyses using PASW Statistics 17 for Windows (SPSS Inc, Chicago, IL). We also investigated the associations using multilevel logistic regression, to take into account the clustering of the children within teams of health care professionals. In addition, the intra-cluster correlation coefficient (ICC) was calculated for weight status of the children to investigate the proportion of the within-cluster variance in the total variance among the children[37]. These analyses were performed using SAS software (version 9.2; SAS Institute Inc, Cary, North Carolina). The ICC appeared to be relatively small (0.01) and the results of the multilevel analyses did not differ significantly from the results of the logistic regression analyses. Therefore, we concluded that the clustering of the children within the teams of health care professionals did not affect the results of this study, and we reported the results of the logistic regression analyses without the multilevel component.

## Results

The prevalence of overweight (obesity included) among the children was 8.8%. Of all included children, mean age was 5.7 (SD 0.4) years, 50.9% were boys, and 13.9% were of non-Dutch ethnicity, 6.5% did not have breakfast daily, 64.3% drank >2 glasses of sweet beverages/day, 6.5% played outside <1 hour/day, and 19.1% watched TV >2 hours/day. In 21.1% of the children, 2 or more of the risk behaviors were present (the sum of the frequencies ‘any 2’ and ‘any 3 or all’). All sociodemographic characteristics of the parents and children, and lifestyle-related characteristics of the children differed statistically significant between the subgroups of children with and without overweight (obesity included), with the exception of sex of the parent who completed the questionnaire, age of the child, and the amount of time the child played outside (Table 1).

**Table 1:** Characteristics of the total study population and according to children’s weight status (n = 7505)

	**Frequency in study population (%) (unless otherwise specified)**			
	**Total**	**Child has overweight (obesity included)**^**a**^		***P*- value**^**b**^
		**No (n = 6847)**	**Yes (n = 658)**	
Parent characteristics				
Mean age, years (SD)	36.8 (4.5)	36.9 (4.5)	36.3 (4.8)	<.01
Mother is respondent	6639 (88.5)	6055 (88.4)	584 (88.8)	.81
Low educational level^c^	1470 (19.6)	1274 (18.6)	196 (29.8)	<.001
Not employed	1888 (25.2)	1699 (24.8)	189 (28.7)	<.05
Single parent	506 (6.7)	437 (6.4)	69 (10.5)	<.001
Child characteristics				
Mean age, years (SD)	5.7 (0.4)	5.7 (0.4)	5.8 (0.4)	.22
Boy	3820 (50.9)	3567 (52.1)	253 (38.4)	<.001
Non-Dutch ethnicity	1044 (13.9)	909 (13.3)	135 (20.3)	<.001
Mean BMI (SD)	15.5 (1.5)	15.2 (1.1)	18.7 (1.4)	<.001
Child risk behaviors				
Having breakfast <7 days/week	488 (6.5)	415 (6.1)	73 (11.1)	<.001
Drinking sweet beverages >2 glasses/day	4826 (64.3)	4377 (63.9)	449 (68.2)	<.05
Playing outside <1 h/day	486 (6.5)	445 (6.5)	41 (6.2)	.79
Watching TV >2 hs/day	1430 (19.1)	1261 (18.4)	169 (25.7)	<.001
Number of child risk behaviors present				
None	2059 (27.4)	1922 (28.1)	137 (20.8)	<.001
Only 1	3686 (51.5)	3530 (51.6)	338 (51.4)	
Any 2	1386 (18.5)	1231 (18.0)	155 (23.6)	
Any 3 or all	192 (2.6)	164 (2.4)	28 (4.3)	

^a^According to the age and sex specific cut-off points for BMI as published by the IOTF [34]

^b^*P*-value for difference between overweight no/yes

^c^Low education level = no education, primary education, lower secondary vocational education, and preparatory secondary vocational education

Compared to the children whom had breakfast every day, children whom did not eat breakfast daily were more likely to have overweight (obesity included) (OR = 1.49, 95% CI: 1.13-1.95, adjusted for confounders). Compared to the children whom watched TV ≤2 hours/day, the OR for having overweight (obesity included) was 1.25 (95% CI: 1.03-1.51, adjusted for confounders) for children whom watched TV >2 hours/day. After including the four behaviors in the model simultaneously, only the association between not having breakfast daily and overweight remained statistically significant (OR = 1.44, 95% CI: 1.09-1.89) (Table 2).

**Table 2:** Associations between the behaviors and overweight (obesity included) among the children (n = 7505)

	**Prevalence of overweight (obesity included)**^**a**^		**OR (95% CI)**	
		***P*-value**^**b**^	**Model 1**	**Model 2**
Having breakfast				
7 days/week	8.3	<.001	1.00	1.00
<7 days/week	15.0		1.49 (1.13 – 1.95)	1.44 (1.09-1.89)
Drinking sweet beverages				
≤2 glasses/day	7.8	<.05	1.00	1.00
>2 glasses/day	9.3		1.17 (0.99-1.40)	1.15 (0.97-1.38)
Playing outside				
≥1 h/day	8.8	.79	1.00	1.00
<1 h/day	8.4		0.98 (0.70-1.37)	0.98 (0.70-1.37)
Watching TV				
≤2 hs/day	8.0	<.001	1.00	1.00
>2 hs/day	11.8		1.25 (1.03-1.51)	1.20 (0.98-1.46)

Model 1: behaviors individually

Model 2: all behaviors included simultaneously

All analyses were adjusted for sex of the child and sociodemographic characteristics (child’s ethnicity, educational level parent, single parenthood, job status of the parent)

^a^According to the age and sex specific cut-off points for BMI as published by the IOTF [34]

^b^*P*-value for difference in prevalence of overweight (obesity included) between child risk behavior not present/present

The number of risk behaviors that were present, was positively associated with having overweight (obesity included), and compared to children with none of the risk behaviors, the OR for having overweight was 1.73 for children with 3 or all behaviors (95% CI: 1.11-2.71, adjusted for confounders) (Table 3).

**Table 3:** Association between number of risk behaviors and overweight (obesity included) (n = 7505)

**Number of child risk behaviors**^**a**^	**OR (95% CI)**
None	1.00
Only 1	1.31 (1.06-1.61)
Any 2	1.48 (1.15-1.89)
Any 3 or all	1.73 (1.11-2.71)

All analyses were adjusted for sex of the child and sociodemographic characteristics (child’s ethnicity, educational level parent, single parenthood, job status of the parent)

^a^The 4 child risk behaviors were 1) playing outside <1 h/day, 2) having breakfast <7 days/week, 3) drinking sweet beverages >2 glasses/day, and 4) watching TV >2 hs/day

## Discussion

We assessed the associations between the four lifestyle-related behaviors having breakfast, drinking sweet beverages, playing outside and watching TV, and overweight among 5-year-old children. The results indicate that not having breakfast daily and spending too much time watching TV are behavioral risk factors for having overweight (obesity included) already at this young age. We also found that not having breakfast every day is a risk factor independent of the other lifestyle-related behaviors. Further, we found that having multiple of the investigated behavioral risk behaviors (not having breakfast daily; drinking >2 glasses of sweet beverages; spending <1 hour playing outside; and spending >2 hours watching TV per day) is associated with an increased risk of having overweight (obesity included) in early childhood.

With the results of our study we further strengthen the literature base regarding the four behavioral risk factors for overweight in early childhood[17-26,38]. We add to the existing knowledge as we included a large study population of young children (n = 7505) with a small age range, so our results could be specifically ascribed to the 5-year-old age group. Further, we included both dietary and physical activity factors, and also took important sociodemographic characteristics like socioeconomic status (SES) and ethnicity into account. We have extended the findings of previous studies by examining how the number of risk behaviors present is associated with the risk of having overweight during early childhood. Although the four risk factors assessed in this study are of practical relevance for guiding well-child visits, we are not aware of the impact of behaviors not included in the study such as consumption of sweet and savory snacks; neither did we include a food frequency questionnaire nor a full assessment of physical activity and sedentary behaviors.

Compared to the data of children/parents with missing data (n = 1279), the population analyzed (n = 7505) included statistically significant less children of non-Dutch ethnicity (*P* < .001), less children with overweight or obesity (*P* < .01), less children with the risk behaviors not having breakfast daily (*P* < .001) and watching TV >2 hours per day (*P* < .001), and more parents with a high educational level (*P* < .001), more parents that were employed (*P* < .001), and less single parents (*P* < .001). Thus, there was some selection towards a study population in which the children more often were of Dutch ethnicity, had a higher SES, had a healthier lifestyle and less often had overweight. So, the prevalence of the risk behaviors and overweight in this study might therefore be somewhat underestimated. However, although we cannot ascertain this, it is unlikely that the associations between risk behaviors and overweight in the study population differ from those in the source population.

Another methodological consideration that needs to be addressed is that the characteristics of the parent and the child were based on self-reported data of the parent, and although anonymity was assured, parents might have given socially desirable answers. Height and weight of the children was, however, measured by trained health care professionals during well-child visits.

In addition to the main logistic regression analyses, in which the behaviors were included as dichotomous variables (risk behavior present or not, based on international recommendations), we also investigated the associations with the behaviors divided into more categories. Overall, the results of these analyses did not differ significantly compared to the results of the main analyses. However, children whom had breakfast less than 5 days a week appeared to have no statistically significant increased risk for having overweight (obesity included). On the other hand, for watching TV the ORs in the fully adjusted model remained statistically significant when watching TV less than 1 hour was used as the reference category (see Additional file 1). Further, we also performed multinomial logistic regression analyses to distinguish between the associations of the risk behaviors with children’s overweight, and their associations with children’s obesity. The association between not having breakfast daily and having overweight remained statistically significant. Watching TV more than recommended appeared to be a risk factor for having obesity, independent of the other lifestyle-related behaviors (see Additional file 2). We also performed an analysis in which we distinguished the subgroup obesity further in obesity versus severe obesity. While there are currently no international BMI cut-off points for severe obesity, we used the following cut-off points based on recent literature on this topic [13] and sample size considerations; for boys ≥20 (kg/m^2^) and for girls ≥21 (kg/m^2^). The results show that watching TV was no longer statistically significant associated with the risk of having obesity (n = 63), but there was a strong association with having severe obesity (n = 63); compared to children whom watched TV ≤2 hs/day, for children whom watched TV >2 h/day the OR was 2.14 (95% CI: 1.26-3.63) (data not shown). On the whole, these findings indicate that there are differences in associations between lifestyle-related behavior and weight status of children for different stages of overweight. We also investigated the associations with the behaviors playing outside and watching TV included in the model as continuous variables. Since these variables did not have a normal distribution, we used the log transformations of these variables. We found that when watching TV increases with a factor of 10, that the risk of having overweight (obesity included) increases with an OR of exp(0.46) (95% CI: 0.20-0.72). For playing outside the increase in OR is exp(0.36) (95% CI: 0.03-0.69) (data not shown). More research is needed to investigate the latter association, as it is not as expected that more time playing outside is associated with a higher risk for having overweight. However, no statistical significant association was found for this behavior in the main or other analyses.

As we used cross-sectional data, the direction of the associations we found can not be confirmed. Spending too much time watching TV might increase the risk for developing obesity, but obese children might also increase the time their watching TV as a consequence of their weight status. For having breakfast, it might be the case that parents let their children skip this meal as a strategy to control the children’s weight, but not much is known from literature about such a mechanism among such young children[39]. It is however more likely that, also considering the age of the children, the skipping breakfast contributed to the excess weight gain and not the other way around. It is known from literature that children whom do not eat breakfast, are more likely to consume unhealthy foods during the day[39], which induces the development of overweight.

In the main logistic regression analyses, we did not found a statistical significant association between the behaviors playing outside or drinking sweet beverages, and the risk for having overweight or obesity at the age of 5 years. An association between these behaviors and having overweight or obesity is likely to appear among the study population when they are getting older. The small average daily energy imbalance that is caused by spending too little time playing outside and drinking too much sweet beverages per day, probably needs to have sustained for several years before an effect on weight can be detected in our study population[17,23]. Further, as also indicated by previous literature, behavioral risk factors tend to cluster together. The association between one behavior and the risk for having overweight might be too weak to appear in statistical analyses, but when multiple of these behaviors are present the effects of these behavioral risk factors might do appear[6,7,13]. We indeed found that with an increasing number of risk behaviors present, the risk for having overweight (obesity included) was also higher. There are several ways in which the four lifestyle-related behaviors might be correlated with and influence one another, and which may also contribute to the apparent increased risk for having overweight when multiple of the behaviors are present. We already mentioned the higher intake of unhealthy, energy dense foods during the day, like sweet beverages, among children whom do not eat breakfast daily[39]. Another mechanism is a potential increase in intake of these unhealthy foods while watching TV or through advertising for these foods, and through food messages embedded within program content[6,7,21,23,40]. Further, the time children spent watching TV might displace time spent in physical activity[6,7].

## Conclusions

Not having breakfast daily and watching TV more than recommended appeared to be risk factors for having overweight (obesity included), already during early childhood. Further, when the number of the risk behaviors (risk behaviors investigated: not having breakfast every day, drinking >2 glasses of sweet beverages, and spending <1 hour playing outside and >2 hours watching TV per day) in these young children increased, also the risk for having overweight increased. This confirms current knowledge among older children that all four risk behaviors are associated with the presence of overweight. It is likely that risk behaviors present in early childhood tend to persist during school age and even adolescence[6,8]. Therefore we recommend studies to develop, implement and evaluate the effectiveness of educational interventions that tackle obesogenic lifestyles during early childhood in order to prevent the onset of overweight and obesity when the children grow up. In the meantime we recommend physicians to target all four behaviors, and especially having breakfast and watching TV, for counseling during well-child visits before adverse habits are established.

## Competing interests

The authors declare that they have no competing interests.

## Author’s contributions

All authors have participated in the concept and design; analysis and interpretation of the data; drafting or revising of the manuscript: HR originated the idea for the study and its design and was responsible for acquiring the grant for the study. LV further developed the described study protocol and was responsible for the data collection, data analyses and reporting of the results of the study. IV helped developing questionnaires, contributed to the data analyses and reporting of the results. LR and AO contributed to the analyses and the reporting of the results. CR and RH contributed to the design of the study, data collection procedures, plan for analysis, and reporting the results. HR supervised the study. All authors provided feedback on the manuscript and approved the final version.

## Supplementary Material

Additional file 1 **Associations between behaviors and overweight, with the behaviors divided in >2 categories (n = 7505).** Description of data: results of the logistic regression analyses with the behaviors of the children divided in more categories than only risk behavior present or not. Click here for file

Additional file 2 **Associations between behaviors and weight status, for subgroups overweight and obesity separately (n = 7505).** Description of data: results of the multinomial logistic regression analyses to distinguish between the associations of the lifestyle-related behaviors with children’s overweight, and their associations with children’s obesity. Click here for file
