# Supplementary material for: Behavioral risk factors for overweight in early childhood; the ‘Be active, eat right’ study
Source: Int J Behav Nutr Phys Act. 2012 Jun 15;9:74. doi: 10.1186/1479-5868-9-74 (PMC3409071; doi:10.1186/1479-5868-9-74)
Supplement: Additional file 1 — Associations between behaviors and overweight, with the behaviors divided in >2 categories (n = 7505). Description of data: results of the logistic regression analyses with the behaviors of the children divided in more categories than only risk behavior present or not. [file 1479-5868-9-74-S1.doc]

Additional file 1 Associations between behaviors and overweight, with behaviors divided in >2 categories (n = 7505)

|  | **Prevalence of overweight (obesity included)^a^** | |  | **OR (95% CI)** | |
| --- | --- | --- | --- | --- | --- |
|  |  | *P*-value^b^ |  | **Model 1** | **Model 2** |
| Having breakfast |  |  |  |  |  |
| 7 days/week (n = 7017) | 8.3 | <.001 |  | 1.00 | 1.00 |
| ≥5 - <7 days/week (n = 317) | 14.2 |  |  | 1.51 (1.08 – 2.10) | 1.47 (1.05 – 2.05) |
| <5 days/week (n = 171) | 16.4 |  |  | 1.45 (0.94 – 2.23) | 1.37 (0.89 – 2.11) |
| Drinking sweet beverages |  |  |  |  |  |
| <1 à 2 glasses/day (n = 306) | 8.2 | .10 |  | 1.00 | 1.00 |
| ≥1 à 2 - <4 à 5 glasses/day (n = 6010) | 8.5 |  |  | 1.09 (0.71 – 1.66) | 1.09 (0.71 – 1.67) |
| ≥4 à 5 - <7 à 8 glasses/day (n = 1062) | 10.3 |  |  | 1.23 (0.78 – 1.95) | 1.18 (0.74 – 1.87) |
| ≥7 à 8 glasses/day (n = 127) | 12.6 |  |  | 1.33 (0.68 – 2.61) | 1.27 (0.65 – 2.51) |
| Playing outside |  |  |  |  |  |
| ≥2 h/day (n = 4185) | 9.5 | .08 |  | 1.00 | 1.00 |
| ≥1 - 2 h/day (n = 2834) | 7.7 |  |  | 0.84 (0.71 – 1.00) | 0.86 (0.72 – 1.03) |
| <1 - ≥0.5 h/day (n = 429) | 8.4 |  |  | 0.93 (0.65 – 1.34) | 0.96 (0.66 – 1.38) |
| <0.5 h/day (n = 57) | 8.8 |  |  | 0.81 (0.32 – 2.07) | 0.87 (0.34 – 2.22) |
| Watching TV |  |  |  |  |  |
| <1 h/day (n = 1743) | 6.3 | <.001 |  | 1.00 | 1.00 |
| ≥1 - <2 hs/day (n = 3985) | 8.7 |  |  | 1.33 (1.06 – 1.66) | 1.32 (1.05 – 1.65) |
| ≥2 - <3 hs/day (n = 1269) | 10.7 |  |  | 1.44 (1.10 – 1.89) | 1.38 (1.05 – 1.81) |
| >3 hs/day (n = 508) | 13.0 |  |  | 1.60 (1.14 – 2.24) | 1.47 (1.04 – 2.07) |

Model 1: behaviors individually

Model 2: all behaviors included simultaneously

All analyses were adjusted for sex of the child and sociodemographic characteristics (child’s ethnicity, educational level parent, single parenthood, job status of the parent)

^a^According to the age and sex specific cut-off points for BMI as published by the IOTF{Cole, 2000 #16}

^b^*P*-value for difference in prevalence of overweight (obesity included) between categories of child behavior
