# Supplementary material for: Behavioral risk factors for overweight in early childhood; the ‘Be active, eat right’ study
Source: Int J Behav Nutr Phys Act. 2012 Jun 15;9:74. doi: 10.1186/1479-5868-9-74 (PMC3409071; doi:10.1186/1479-5868-9-74)
Supplement: Additional file 2 — Associations between behaviors and weight status, for subgroups overweight and obesity separately (n = 7505). Description of data: results of the multinomial logistic regression analyses to distinguish between the associations of the lifestyle-related behaviors with children’s overweight, and their associations with children’s obesity. [file 1479-5868-9-74-S2.doc]

Additional file 2 Associations between behaviors and weight status, for subgroups overweight and obesity separately (n = 7505)

| **Overweight (n = 532)** | | | | | |
| --- | --- | --- | --- | --- | --- |
|  | **Prevalence of overweight^a^** | |  | **OR (95% CI)** | |
|  |  | *P-*value^b^ |  | **Model 1** | **Model 2** |
| Having breakfast |  |  |  |  |  |
| 7 days/week | 6.9 | <.001 |  | 1.00 | 1.00 |
| <7 days/week | 11.9 |  |  | 1.46 (1.07-1.97) | 1.42 (1.05-1.93) |
| Drinking sweet beverages |  |  |  |  |  |
| ≤2 glasses/day | 6.4 | <.05 |  | 1.00 | 1.00 |
| >2 glasses/day | 7.7 |  |  | 1.19 (0.98-1.44) | 1.17 (0.97-1.42) |
| Playing outside |  |  |  |  |  |
| ≥1 h/day | 7.2 | .94 |  | 1.00 | 1.00 |
| <1 h/day | 7.3 |  |  | 1.03 (0.72-1.47) | 1.03 (0.72-1.47) |
| Watching TV |  |  |  |  |  |
| ≤2 hs/day | 6.8 | <.01 |  | 1.00 | 1.00 |
| >2 hs/day | 9.0 |  |  | 1.14 (0.92-1.42) | 1.10 (0.88-1.37) |
| **Obesity (n = 126)** | | | | | |
|  | **Prevalence of obesity^a^** | |  | **OR (95% CI)** | |
|  |  | *P*-value^b^ |  | **Model 1** | **Model 2** |
| Having breakfast |  |  |  |  |  |
| 7 days/week | 1.7 | <.001 |  | 1.00 | 1.00 |
| <7 days/week | 3.9 |  |  | 1.60 (0.93-2.73) | 1.48 (0.86-2.54) |
| Drinking sweet beverages |  |  |  |  |  |
| ≤2 glasses/day | 1.6 | .32 |  | 1.00 | 1.00 |
| >2 glasses/day | 1.9 |  |  | 1.13 (0.77-1.66) | 1.07 (0.73-1.58) |
| Playing outside |  |  |  |  |  |
| ≥1 h/day | 1.8 | .43 |  | 1.00 | 1.00 |
| <1 h/day | 1.3 |  |  | 0.76 (0.33-1.75) | 0.77 (0.34-1.78) |
| Watching TV |  |  |  |  |  |
| ≤2 hs/day | 1.4 | <.001 |  | 1.00 | 1.00 |
| >2 hs/day | 3.4 |  |  | 1.71 (1.16-2.53) | 1.65 (1.11-2.44) |

Reference category is ‘no overweight’

Model 1: behaviors individually

Model 2: all behaviors included simultaneously

All analyses were adjusted for sex of the child and sociodemographic characteristics (child’s ethnicity, educational level parent, single parenthood, job status of the parent)

^a^According to the age and sex specific cut-off points for BMI as published by the IOTF[33]

^b^*P*-value for difference in prevalence of overweight/obesity between child risk behavior not present/present
